# Clinical outcomes of epithelial ingrowth following laser *in situ* keratomileusis with mechanical debridement and compressed heating air fow: a case series

**DOI:** 10.5935/0004-2749.20230031

**Published:** 2023

**Authors:** Patrick Frensel Tzelikis, Guilherme Victor Alves, Guilherme Rocha, Canrobert Oliveira

**Affiliations:** 1 Department of Refractive Surgery, Hospital Oftalmológico de Brasília, Brasília, DF, Brazil

**Keywords:** Epithelium/growth & development, Endothelium, corneal, Corneal diseases, Keratomileusis, laser *in situ*, Photorefractive keratectomy, Refractive surgery, Visual acuity, Epitélio/crescimento & desenvolvimento, Endotélio corneano, Doenças da córnea, Ceratomileuse assistida por excimer laser *in situ*, Ceratectomia fotorrefrativa, Procedimentos cirúrgicos refrativos, Acuidade visual

## Abstract

**Purpose:**

To describe the clinical outcomes of manual scraping of epithelial ingrowth followed by compressed heating air flow after laser *in situ* keratomileusis (LASIK).

**Methods:**

We underwent a retrospective, noncomparative, and interventional case series. Twenty eyes of 17 patients were included in this study. Each patient with a history of LASIK underwent epithelial removal with mechanical debridement followed by compressed heating air flow. Our primary outcome was the recurrence of epithelial ingrowth after 3 months of follow-up, while our secondary outcomes were uncorrected distance visual acuity, corrected distance visual acuity, and complications after surgery.

**Results:**

Ten patients (58.8%) were male, and eight eyes of seven (41.2%) patients underwent primary LASIK surgery, while12 eyes of 10 patients had flap-lift retreatment LASIK; sixteen eyes (80.0%) underwent mechanical microkeratome LASIK and four (20.0%) underwent femtosecond laser-assisted LASIK. Mean age at surgical removal of epithelial ingrowth was 37.0 years ± 9.3 years (range 24 to 55 years). There was recurrence of ingrowth in two eyes (10%) after 3 months of follow-up. The mean corrected distance visual acuity of patients before surgery was 0.07 ± 0.09 logMAR, and after the last follow-up was 0.02 ± 0.04 logMAR (p=0.06). The odds ratio of presenting with epithelial ingrowth after LASIK enhancement compared to primary LASIK was 29.41.

**Conclusion:**

Manual scraping followed by compressed heating air flow is a safe and effective treatment of clinically significant epithelial ingrowth after LASIK. At the last follow-up, no eye lost any line in corrected distance visual acuity.

## Abstract (pt)

**Objetivo:**

Descrever os resultados clínicos do tratamento do crescimento epitelial através da técnica de remoção manual seguido da utilização de um compressor de ar comprimido aquecido após a cirurgia de *laser in situ keratomileusis* (LASIK).

**Métodos:**

Vinte olhos de 17 pacientes foram incluídos no estudo. Cada paciente havia sido submetido a cirurgia de LASIK com presença de crescimento epitelial e foi submetido a tratamento cirúrgico para sua retirada. O objetivo primário foi identificar a presença de crescimento epitelial recorrente ao final de 3 meses de seguimento. Os objetivos secundários foram as medidas de acuidade visual sem correção, acuidade visual com correção, e complicações pós-operatórias.

**Resultados:**

Dez pacientes (58,8%) eram homens e 7 mulheres. Oito olhos de sete (41,2%) pacientes apresentavam cirurgia de LASIK primária e 12 olhos de 10 pacientes tinham cirurgia de LASIK com retratamento; dezesseis olhos (80%) utilizaram microcerátomo manual e quatro (20%) laser de femtosegundo. A média de idade no momento da cirurgia de remoção do epitélio era de 37,0 anos ± 9,3 (DP) (variando de 24 a 55 anos). Ocorreu recidiva do crescimento epithelial em dois olhos (10%) após 3 meses de seguimento. A acuidade visual sem correção antes da cirurgia era de 0,07 ± 0,09 logMAR, e após a cirurgia passou para 0,02 ± 0,04 logMAR (p=0,06). A chance (odds ration) de aparecimento do crescimento epithelial após uma reoperação de LASIK é 29,41 vezes maior do que no LASIK primário.

**Conclusão:**

A técnica de remoção epitelial manual seguida da utilização de ar comprimido aquecido é segura e efetiva no tratamento do crescimento epitelial após LASIK. Ao final do último acompanhamento, nenhum olho apresentou perda de linhas de visão.

## INTRODUCTION

Epithelial ingrowth remains a relevant topic in modern refractive surgery. It leads to unsatisfying results from a continuous foreign-body sensation, irregular astigmatism, decrease visual acuity, and flap melting. Its incidence varies in the literature from 0% to 20% following laser *in situ* keratomileusis (LASIK) cases^(1,2,3,4)^. Although epithelial ingrowth is frequently self-limiting and can be observed without requiring intervention, in a small number of cases epithelial ingrowth may progress with permanent visual loss^(5)^.

Two mechanisms related to epithelial ingrowth appearance have been described: the first one is the deposition of epithelial cells by microkeratome or other devices during the surgical procedure. The second mechanism is the postoperative migration of epithelial cells from the flap gutter across the flap interface due to the presence of a pathway. Risk factors are mechanical microkeratome, hyperopic LASIK treatment, buttonholes, retreatments, and advanced age^(6,7)^.

A number of different techniques for removing epithelial ingrowth have been described. These include surgical lifting and debridement of the LASIK flap^(8)^, ethanol-assisted debridement^(9)^, flap suturing^(10)^, Neodymium:YAG laser treatment^(11)^, and fibrin glue surgical adhesive^(12)^. Despite diverse treatment options available, recurrence of epithelial ingrowth is frequent with rates ranging from 5% to 68% after surgical removal^(6,7,8,9,10,11,12,13,14)^.

In this study, we presented 20 cases of flap lifting and manual scraping of the epithelial ingrowth combined with compressed heating air flow after LASIK seen at the Brasília Ophthalmologic Hospital (HOB), Brasília, Brazil, between January 2014 and December 2017.

## METHODS

We underwent a retrospective, noncomparative, interventional case series. All cases of epithelial ingrowth after LASIK who underwent flap-lift and epithelial debridment in the HOB were identified from the records of the surgeons performing regular LASIK between January 2012 and May 2017.

We included those who underwent post-LASIK epithelial ingrowth significant enough to warrant removal. Clinically significant epithelial ingrowth was defined as an ingrowth greater than 2.0 mm centrally from the flap edge or significant foreign-body sensation related to epithelial irregularity.

The study was performed according to established ethical standards for clinical research Institutional Review Board of the Brasília Ophthalmologic Hospital in Brazil. Demographic and surgical details were recorded, including baseline characteristics of patients, the indication for epithelial ingrowth removal, time from LASIK to epithelial ingrowth; extent and location of epithelial ingrowth; risk factors for ingrowth and the visual outcome before and after surgical repair. Primary outcome measures included the recurrence of epithelial ingrowth after 3 months of follow-up. Secondary outcomes included uncorrected distance visual acuity (UDVA), corrected distance visual acuity (CDVA), and complications 3 and 6 months after surgery.

Primary laser *in situ* keratomileusis was performed with either a Hansatome microkeratome (Chiron Corp, Irvine, California, USA) or a Femtosecond Intralase. The 120-micro plate of the Hansatome was used to create a superiorly hinged laser *in situ* keratomileusis flap; the femtosecond laser (Intralase Corporation, Advanced Medical Optics, Inc, CA) was programmed to delivered the following settings: 100 µm thickness; 9.0 mm diameter, superior hinge with 45-degree angle, and 60-degree side-cut angle. In all eyes, surgery was planned to leave minimum 30 microns of residual stromal thickness using the above settings.

### Surgical technique

The same surgeon performed all the procedures. The cornea was marked with a radial marker with Rose Bengal ink. The flap edge was determined by depression with a spatula, noting the change in light reflex. The corneal epithelium was removed over the 1.0-mm margin of the flap on either side of the gutter to allow adherence of the flap after removal of the ingrowth. The flap was then lifted using the same spatula. Epithelial cells were removed with a blunt spatula, and a dry lint-free sponge from behind the flap and the underlying stromal bed. The flap was laid back down after irrigation with a balance salt solution, stretched, and positioned in place with a delicate moinstened brush. After, a local compressed heating air flow was directed toward the cornea to dry and promote flap adherence for 30 seconds in a temperature of 40°F. A bandage contact lense was placed. Moxifloxacin (Vigamox) and ketorolac of tromethamina 0.4% (Acular LS) were given immediately postoperatively and continued thrice daily until removal of the contact lens.

### Postoperative

Each eye was evaluated on the first day after surgery and after 7 days for removal of the bandage contact lens. The presence or absence of epithelial ingrowth was evaluated again after 3 months and at the final follow-up.

All data analyses were performed using SPSS statistical program (version 17.0, SPSS, Inc, Chicago, Illinois, USA). Quantitative data were described as mean ± standard deviation (range). Mann-Whitney test was employed to compare pre and postoperative visual acuity. The 2-tailed chi-square test (χ^2^) or Fisher exact test were used for statistical analysis of categorical variables. Differences were considered statistically significant when the p-value was less than 0.05.

## RESULTS

The review comprised 1854 cases of LASIK during the 5-year study period. The incidence of epithelial ingrowth after primary LASIK in the present study was 0.43% (8 eyes). The incidence after enhancement (flap-lift retreatment LASIK) was 12.6%. The incidence of epithelial ingrowth was significantly higher in the enhancement group (OR, 0.034; p<0.001). The corneal flap was created with Hansatome microkeratome in 16 eyes (80%) and by femtosecond laser in 4 eyes (20%).

The study included 20 eyes of 17 patients, 10 (58.8%) were men and 7 were women. The mean patient age at the time of epithelial ingrowth treatment was 37 ± 9.3 years (SD) (range 24 to 55 years). Median duration between LASIK and epithelial ingrowth treatment was 85.1 ± 98 days (range, 15 days to 270 days). The epithelial ingrowth was detected at the 1-month postoperative visit in 12 eyes (60%). The mean time between the primary procedure and the enhancement was 103 ± 50.2 days (range, 33 to 180 days).

**Figure 1:**
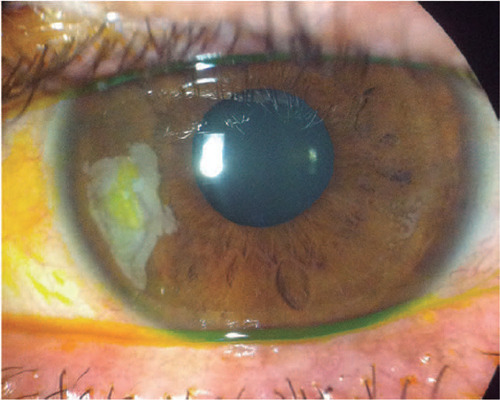
Slit lamp photograph demonstrates epithelial ingrowth in the left eye in a patient with chronic foreign-body sensation

All eyes were diagnosed of clinically significant epithelial ingrowth, and the most common indications for the epithelial ingrowth were corneal irregularities causing symptoms such as halos, glare, or reduced visual acuity, and chronic foreign-body sensation (Figure 1). No patient had diabetes or anterior basement membrane dystrophy. All cases had peripheral ingrowth with inward contiguous extensions toward the visual axis.

All cases were followed up for at least 6 months. The mean follow-up time after the repair surgery was 18 ± 16 months (range, 6 to 61 months). None of the cases had a loss of CDVA over the pretreatment measurement. Two eyes (10%) exhibited mild recurrence epithelial ingrowth. These patients did not require subsequent treatment.

The mean preoperative logMAR UDVA was 0.13 ± 0.10 (range 0.00 to 0.30). The mean logMAR UDVA was 0.10 ± 0.19 (range, 0.00 to 0.30) at the last follow-up. The mean logMAR CDVA was 0.07 ± 0.09 (range, 0.00 to 0.30) before epithelial cleaning, and 0.02 ± 0.04 (range, 0.00 to 0.10) after the procedure. Before treatment, 12 eyes (60%) had 0.00 logMAR or better CDVA, 16 eyes (80%) had 20/25 or better CDVA, and 20 eyes (100%) had 20/40 or better CDVA. At the last follow-up after the procedure, 16 eyes (80%) had 0.00 logMAR or better CDVA, and 20 eyes (100%) had 0.10 logMAR or better CDVA. The difference between the mean CDVA before the procedure and at the last visit after surgery was not significant (p=0.15).

At the last follow-up, there were no associated complications during or after the procedure. None of the eyes lost any line in CDVA, developed epithelial defect, diffuse lamellar keratitis, or infections.

## DISCUSSION

Epithelial ingrowth is an unusual complication after laser *in situ* keratomileusis (LASIK). In this study, the incidence of epithelial ingrowth after primary LASIK was 0.43% in 1854 eyes and was consistent with literature where the incidence ranged from 0% to 20%^(1,2,3,4)^. This wide difference in the reported incidences could be explained by the diverse methodology employed in the studies. For example, some included asymptomatic and nonprogressive cases, and others not. In addition, this variation can also be related to the definition of epithelial ingrowth or to the method used to lift and replace the LASIK flap during the procedure^(15,16)^. Technically, the presence of any epithelial cells beneath the flap after LASIK can be termed epithelial ingrowth as they do not belong to this site.

Many risk factors described in the literature could lead to the development of epithelial ingrowth. These include hyperopic LASIK correction, intraoperative epithelial defects^(17)^, thinner corneal flaps^(18)^, epithelial basement membrane dystrophy^(19)^, use of bandage contact lens^(13)^, presence of diabetes mellitus^(6)^, trauma^(5,6,7,17)^, recurrent corneal erosions^(17)^, older patient age^(13)^, surgeon inexperience^(6)^, and LASIK enhancement^(3,4,5,6,7)^.

Enhancement procedures also have an increased risk of epithelial ingrowth. In this study, the incidence of epithelial ingrowth was 29 times greater for enhancement procedures than for primary LASIK. In our series of LASIK, we observed a 12% incidence in a sample of 95 eyes of epithelial ingrowth after retreatment. Chan et al. reported as much as 40% of cases with epithelial ingrowth after retreatment. Mulhern et al.^(20)^ reported an incidence of epithelial ingrowth in 26% and 44% for flap-lift after myopic and hyperopic retreatments, respectively. Pérez-Santonja et al.^(14)^ revealed a 23.5%-increase in ingrowth after myopic retreatment. Caster et al.^(1)^ revealed that 2.3% of clinically significant epithelial ingrowth was observed after flap-lift retreatment LASIK. Kamburoglu and Ertan^(2)^ revealed an incidence of 1.8% in 108 enhancement cases after femtosecond laser-assisted LASIK.

Usually, the epithelial ingrowth is detected by the 1-month postoperative visit. In our series, this was detected in 12 eyes (60%). Wang and Maloney^(3)^ revealed that 90% of cases were detected within 2 months after surgery. Early recognition allows for appropriate monitoring and intervention when required. Most cases were self-limited around the edge of the flap and required no. However, Naoumidi et al.^(21)^, suggested that epithelial ingrowth should be treated as soon as possible to prevent progression, irregular astigmatism, and stromal melting.

Treatment of epithelial ingrowth is controversial within different procedures such as lifting the flap and scraping the epithelial, with or without adjunctive treatments such as mitomycin, alcohol application, phototherapeutic keratectomy, amniotic membrane, suturing of the flap, or fibrin glue application^(11,12,13,14,15,16)^. We treated epithelial ingrowth by lifting the LASIK flap, carefully scraping the stroma bed and the posterior surface of the flap, with careful irrigation of the interface, precise repositioning of the flap, and a local compressed heating air flow directed toward the cornea to dry and promote flap adherence for 30 seconds in a temperature between 100-120 fahrenheit (F). Epithelial ingrowth recurred in 2 (10%) eyes following this approach. The most common method of treating epithelial ingrowth has been primary lifting and scraping the epithelium from the back of the flap and stroma. Wang and Maloney^(3)^ found a 44% recurrence rate of epithelial ingrowth and a 23% rate of clinically significant recurrence with the use of manual scraping alone. Although our technique is similar to the most used techniques for treating epithelial ingrowth, the use of locally compressed heating air flow contributed to a greater adhesion of the flap and consequently less risk of recurrence.

Other reports have found that epithelial ingrowth treatment can be associated with a good visual outcome, depending upon the nature and severity of ingrowth. Henry et al.^(22)^, reported a final UDVA of 20/20 or better in 45% of eyes and 20/40 or better in 80% of eyes. Our series supports this finding. The difference between the mean BCVA at the last visit before treatment and the last visit after the repair surgery was not significant (p=0.15).

Our study had limitations. First, sample size of the study was small to produce significant results. Further analysis should include more patients and compare different procedures of epithelial ingrowth treatment, as well as elucidate the real benefit of our technique. Second, this was a retrospective study: thus, the risk of inaccurate data and the control the investigator over the approach to sampling and follow-up of the participants were not optimal and ideal.

In summary, we found a higher incidence of epithelial ingrowth after LASIK enhancement. The odds ratio of presenting with epithelial ingrowth after LASIK enhancement compared to primary LASIK was 29.41. This study also found that lifting the LASIK flap, carefully scraping the stroma bed and the posterior surface of the flap, and using a local compressed heating air flow is an easy, safe, and effective technique for the treatment of epithelial ingrowth after LASIK surgery. Thus, we recommend the undertaking of a prospective study be conducted to compare different techquines for treating patients with epithelial ingrowth.
